# A cross-talk between p16^High^ senescence and cellular reprogramming

**DOI:** 10.1042/CS20260240

**Published:** 2026-05-29

**Authors:** Alexander Emelyanov, Dmitry V. Bulavin

**Affiliations:** Institute for Research on Cancer and Aging of Nice (IRCAN), Université Côte d'Azur, INSERM, CNRS, Nice, France

**Keywords:** induced pluripotent stem cells, p16, partial reprogramming, reprogramming, senescence

## Abstract

Cellular senescence and OSKM (Oct4, Sox2, Klf4, and Myc)-mediated reprogramming represent interconnected biological programs that both play important roles in regulating cellular plasticity. Recent studies have highlighted the role of p16-driven senescence in establishing a stable barrier to reprogramming by limiting epigenetic flexibility. Mechanistically, p16^High^ senescent fibroblasts enforce this barrier through stress-induced and AP-1-driven epigenetic remodeling and NNMT-mediated metabolic SAM depletion, which restricts methylation-dependent chromatin remodeling in both p16^High^ and neighboring p16^Low^ cells via a paracrine mechanism. Conversely, clearance of p16^High^ cells restores SAM levels and enhances cellular plasticity in neighboring cells, enabling the acquisition of totipotent-like states during reprogramming. Within p16^High^ cells themselves, reprogramming can reverse some features of senescence, restoring more youthful cellular states under controlled conditions. Importantly, p16^High^ cells remain highly resistant to full reprogramming, minimizing the risk of teratoma and tumor formation *in vivo* and making them promising target for rejuvenation strategies based on partial reprogramming. In this review, we examine the molecular interplay between p16^High^ senescence and reprogramming, highlighting their dual roles as both barriers to and facilitators of cell fate transitions.

Cellular reprogramming refers to the process by which differentiated somatic cells reacquire developmental plasticity and revert toward a pluripotent or more youthful cellular state. Experimentally, this is commonly achieved through expression of the Yamanaka factors OSKM (Oct4, Sox2, Klf4, and c-Myc), a transcription factor combination capable of inducing pluripotent stem cells (iPSCs) from differentiated cells [1,2]. Cellular senescence is a stress- and DNA damage-induced cell state characterized by stable cell-cycle arrest, chromatin remodeling, metabolic rewiring, and active communication with the surrounding tissue through the senescence-associated secretory phenotype (SASP) [3,4]. Beyond classical soluble SASP factors, senescent cells signal through extracellular matrix remodeling, receptor-mediated interactions, and extracellular vesicles. While senescence has historically been viewed as a tumor-suppressive mechanism, it is now increasingly recognized as a dynamic cellular state contributing to tissue homeostasis, aging, and regenerative capacity [5–7]. Cellular senescence and OSKM-mediated reprogramming, therefore, represent interconnected biological programs that both play important roles in regulating cellular plasticity.

An important conceptual advance in recent years has been the recognition that senescence is not uniform. In particular, accumulating evidence suggests that the p16^High^ (CDKN2A/INK4A) cellular state represents a senescence program that is qualitatively distinct from other forms, such as p21^High^ senescence [8–11]. For this reason, here we focus only on the role of p16^High^ senescence in reprogramming, while highlighting possible differences with other senescent cell types.

Use of defined genetic lineage tracing mouse models showed that p16^High^ senescence and cellular reprogramming engage in a bidirectional and context-dependent cross-talk that shapes both reprogramming efficiency and attainable cell fate states [9,12,13]. Rather than representing a terminal, stress-induced cell state that accompanies reprogramming process, emerging evidence supports the notion that p16^High^ senescence contributes to remodeling the molecular environment in which reprogramming unfolds, ultimately influencing transcriptional dynamics, chromatin accessibility, and paracrine signaling. This in turn reduces reprogramming efficiency both *in vitro* and *in vivo* [9,14,15].

In this review, p16^High^ senescence is positioned as an important gatekeeper of plasticity during OSKM-induced full and partial reprogramming, and the base mechanistic framework that accompanies p16^High^-dependent control of cellular plasticity is discussed.

## p16^High^ senescence as a cell-intrinsic and a non-cell-autonomous modulator of plasticity

Single-cell transcriptomic and lineage-tracing studies demonstrate that p16^High^ cells constitute a constrained cellular population that is refractory to OSKM-induced reprogramming *in vitro* and teratoma formation *in vivo* [9]. These cells most likely stall at abortive reprogramming intermediates that subsequently fail to contribute to iPSC colonies. As a result, cells that enter a p16^High^ state are largely excluded from productive reprogramming trajectories and fail to transition toward pluripotent state [9]. Importantly, this resistance cannot be explained by cell-cycle arrest alone. The inability to reprogram p16^High^ cells may help explain a long-standing paradigm: while the removal of proliferative barriers (e.g., via RB or p53 modulation) can enhance reprogramming efficiency, it is insufficient to fully restore cellular plasticity in aged or senescent cells [9,14–16]. p16^High^ senescence thus represents a qualitative change in cell state, rather than merely a quantitative reduction in proliferative capacity.

A defining feature of p16^High^ senescence is epigenetic entrenchment rather than simple chromatin closure. Senescent cells exhibit extensive remodeling of chromatin accessibility landscapes, with enrichment of AP-1-dominated enhancers and depletion of developmental and pluripotency-associated regulatory regions. Chronic activation of AP-1 factors (e.g., JUN, FOS, and FRA1), driven by DNA damage, stress-induced, and inflammatory signaling, creates a competitive transcriptional environment that potentially may actively sequester reprogramming factors away from productive binding sites [17,18]. In this context, OSKM factors must extinguish stress-responsive enhancer landscapes and redistribute transcription factor occupancy toward developmental and pluripotency-associated enhancers [19–23]. In p16^High^ senescent fibroblasts, persistent stress-induced and AP-1-driven transcriptional activity is likely to attenuate this redistribution, providing a mechanistic explanation for why sustained inflammatory and mesenchymal gene programs strongly predict reprogramming failure, as these programs are commonly overrepresented in trajectories that stall early during OSKM reprogramming [24]. This framework could also potentially link senescence to the recently described phenomenon of mesenchymal drift during aging, which could be reversed by partial reprogramming prior to dedifferentiation [24]. Thus, at the cell-intrinsic level, induction of p16^High^ senescence enforces a stable anti-plasticity program that may antagonize reprogramming factor activity.

Metabolic inflexibility is another reinforcing layer of senescence-imposed plasticity restriction. p16^High^ cells exhibit mitochondrial dysfunction, altered NAD+/NADH ratios, and chronic mTOR activation, limiting the availability of metabolites such as acetyl-CoA and α-ketoglutarate required for histone acetylation and TET-mediated DNA demethylation [25–28]. Partial reprogramming and senomorphic interventions can partially restore metabolic flexibility, indirectly enhancing epigenetic plasticity; yet this may not be sufficient in p16^High^ senescent cells.

**Figure 1:**
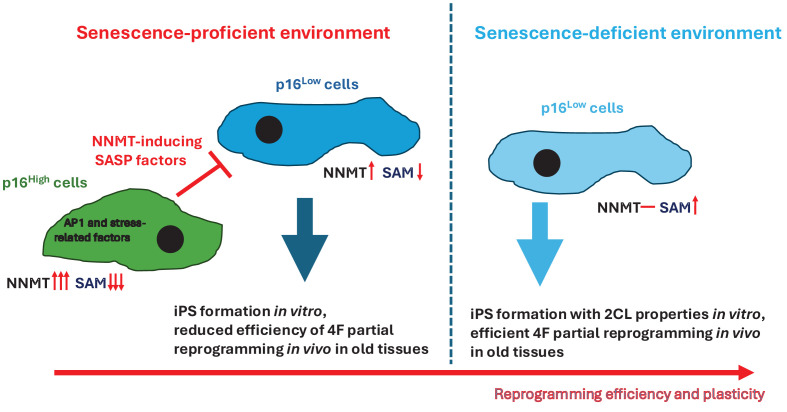
A role for p16^High^ senescence in cellular reprogramming and plasticity Schematic representation of how senescence-competent and senescence-deficient environments differentially influence cellular plasticity and reprogramming efficiency. In a senescence-proficient context, induction of stress-associated and AP-1-related factors in p16^High^ cells produces SASP that regulates NNMT expression and alters SAM availability, ultimately limiting reprogramming efficiency. This environment is associated with reduced efficiency of 4-factor (4F)-mediated partial reprogramming *in vivo*, particularly in aged tissues, and decreased iPSC formation *in vitro*. In contrast, in a senescence-deficient environment, the absence of p16^High^ cells favors enhanced NNMT/SAM balance, promoting increased cellular plasticity, improved iPSC formation with 2-cell-like (2CL) features *in vitro*, and more efficient 4F-induced partial reprogramming *in vivo*.

Our recent mechanistic insights further reveal a metabolic layer in p16^High^ senescent fibroblasts that shows elevated NNMT activity, reduced intracellular SAM availability, and thereby constrains methylation-dependent epigenetic remodeling (Figure 1) [9]. Together with the metabolic changes described above, a reduced capacity to actively engage methylation due to lower SAM levels could further explain the lack of reprogramming in p16^High^ fibroblasts. Furthermore, in mixed cultures, p16^High^ fibroblasts, via secretion of different cytokines and chemokines, could control NNMT expression in neighboring p16^Low^ cells via activation of the JAK/STAT3 signaling pathway with IL-6 family (gp130) cytokines such as IL6, IL11, oncostatin M, and leukemia inhibitory factor; other interleukins such as IL10, IL21, IL31, and IL27; several colony-stimulating and hematopoietic factors such as granulocyte, granulocyte-macrophage, and macrophage colony-stimulating factors erythropoietin; hormones like leptin; and some growth factors [12,13,29]. Such a large spectrum of factors that can induce NNMT provides high robustness to p16^High^ senescence-driven changes (Figure 1). This, in turn, results in reduced levels of SAM, constraining methylation-dependent epigenetic changes in reprogrammable neighboring p16^Low^ cells, ultimately reducing their plasticity. In turn, removal of p16^High^ cells during reprogramming restores SAM levels, enabling extensive chromatin reconfiguration and facilitating access to highly plastic, 2C/totipotent-like transcriptional states [9]. Thus, senescence does not merely block reprogramming through checkpoint enforcement but also rewires metabolic-epigenetic coupling to limit cell fate transitions.

p16^High^ senescent cells emerge early during reprogramming and reduce the efficiency of iPSC formation in neighboring cells *in vitro*; however, the *in vivo* situation is somewhat different. Using a lineage tracing model to follow the fate of p16^High^-expressing cells during *in vivo* reprogramming, we recently found that emerging p16^High^ PDGFRα-positive mesenchymal cells appear only when NANOG-positive reprogrammed cells are established, and thus this senescence program is largely excluded from initial reprogramming events *in vivo* in young mice [9]. Furthermore, p16^High^ PDGFRα-positive fibroblasts that emerge once iPSCs begin to differentiate into different lineages, via paracrine signaling, negatively affect teratoma growth and reduce the plasticity of reprogrammed iPSCs, ultimately attenuating their acquired developmental potential. In turn, removal of p16^High^ cells *in vivo* allows the maintenance of a higher level of plasticity in reprogrammed cells, which is accompanied by the appearance of additional extra-embryonic lineages that are largely absent from teratomas from wild-type mice [9].

It is important to note that several studies have proposed that senescent cells can facilitate reprogramming through paracrine mechanisms [29,30]. *In vitro*, conditioned medium from senescent cells enhances OSKM-mediated reprogramming via SASP signaling, with IL-6/STAT3 identified as a key mediator. In this context, IL-6 may function analogously to LIF by activating STAT3 signaling, although its ability to further enhance reprogramming in the presence of saturating levels of LIF remains unclear. *In vivo*, the pro-reprogramming effects of senescent cells appear to be particularly relevant in settings of tissue injury and inflammation. Although the precise lineage of the contributing senescent cells has not been definitively established, they likely arise from multiple sources, including p21^High^ senescent cell populations as well as p16^High^ senescence-like immune cells. These cells can secrete SASP factors that may be fundamentally distinct from those produced by p16^High^ senescent fibroblasts [9,11]. Thus, under conditions of tissue injury, certain senescent and senescence-like cells may act as transient signaling hubs that lower reprogramming barriers in neighboring cells.

Importantly, the role of inflammation and tissue damage *in vivo* may be particularly relevant during later stages of reprogramming, when a mild and transient inflammatory response can be beneficial independently of senescent cells. Such signaling activates pathways including NF-κB and PI3K-AKT, which can transiently stimulate mTOR activity and support the increased metabolic and biosynthetic demands of emerging pluripotent cells [31]. However, successful completion of reprogramming requires that this inflammatory response subsequently resolve. Only a transient inflammatory burst, rather than sustained signaling, appears permissive for stable acquisition of pluripotency, as chronic inflammation can lead to persistent mTOR activation, impaired autophagic clearance required for somatic identity erasure, increased cellular damage, and failure to properly stabilize induced pluripotent stem cells [32,33]. In this context, it is also important to consider that sensitivity to senolytic interventions may not strictly correlate with canonical senescence markers such as p16 or p21, since senolytic agents primarily target stress-adaptive pro-survival pathways, including BCL-2 family signaling, which may also be transiently engaged in activated or metabolically stressed non-senescent cells [34–36]. Therefore, transient inflammation during tissue injury may facilitate cellular plasticity and reprogramming, whereas chronic inflammation or persistence of the p16^High^ senescent state acts as a barrier to successful completion of reprogramming. This contrasts with the role of p16^High^ senescent fibroblasts during steady-state *in vivo* reprogramming, where they primarily function as a barrier to cellular plasticity [9].

An additional important technical point should be considered when interpreting experiments involving the use of senolytic drugs that target senescent cells [9,29]. We observed that standard concentrations of commonly used senolytics markedly impair reprogramming efficiency at very early stages *in vitro*, prior to the detectable emergence of any senescent cells. This indicates that, in some cases, the observed reduction in reprogramming when senolytic drugs are used cannot be unequivocally attributed to senescent cell clearance [9]. Therefore, careful dose titration, rigorous temporal analysis, and appropriate controls are essential to distinguish genuine senescence-dependent effects from direct pharmacological interference with the reprogramming process. Failure to account for these factors may lead to misleading mechanistic conclusions.

The dual role of senescent cells in reprogramming, under normal conditions or in the presence of strong inflammatory or tissue damage/injury signals, highlights the importance of timing and context. Acute, transient p21^High^ senescence as well as induction of senescence-like immune cells can potentially support regeneration and reprogramming of neighboring cells under certain conditions. In contrast, the accumulation of exclusively p16^High^ senescent PDGFRα-positive mesenchymal cells, including fibroblasts, shifts the balance toward impaired plasticity. Therefore, effective rejuvenation strategies must selectively bypass or remodel the senescence-imposed barrier without unleashing uncontrolled dedifferentiation. This is particularly important in the context of rejuvenation using partial OSKM reprogramming in aged organisms.

Since p16^High^ cells, as well as other senescent and senescence-like subtypes, accumulate in the aging organism, the natural question arises as to whether they overall favor or suppress reprogramming in such a complex environment. Our own analysis showed that mice already at middle age (1 year) exhibit a dramatic drop in the capacity to develop teratomas under continuous, ubiquitous, and moderate induction of OSKM [9]. While the impact of extreme age (2 years and more) still needs careful assessment, one contributing factor is the accumulation of numerous somatic mutations during aging. In turn, some mutations could significantly facilitate the reprogramming process, which could lead not to true teratoma formation, but rather to cancer-related pathologies, leaving an impression of accelerated reprogramming [37]. Thus, the assessment of reprogramming efficiency in old mice remains to be carefully evaluated. So far, most data point to a suppression of reprogramming in aged mice, which in part could be due to accumulation of p16^High^ senescent cells, with further possibility of tumor formations after reprogramming of old cells carrying significant mutation burden.

Collectively, p16^High^ senescence is positioned as both a consequence and a regulator of cellular reprogramming. Productive reprogramming requires either avoidance, reversal, or bypass of the p16^High^ state, while the timing, duration, and clearance of senescent cells determine whether senescence-associated programs constrain or transiently support plasticity.

## p16^High^ senescence and access to totipotent-like states during reprogramming

Recent genetic and chemical dissection of senescent cell contributions to iPSC reprogramming provides critical mechanistic depth to the p16^High^ framework [9,12,13]. Selective removal of p16^High^ senescent cells markedly accelerates *in vivo* OSKM-induced reprogramming, consistent with observations in p53-deficient mice [13]. Furthermore, iPSC lines emerging in senescence-deficient contexts exhibit transcriptional and epigenetic features characteristic of 2CLCs and experimental totipotency, representing the highest state of cellular plasticity. As discussed above, this demonstrated that p16^High^ senescence acts as an active brake on reprogramming kinetics and cell fate plasticity [9,30].

Entry into the embryonic 2-cell totipotent stage represents a critical developmental transition associated with zygotic genome activation (ZGA). This state is marked by a significant increase in the expression of multiple families of transposable elements (TEs), which constitute a strong and important force for the creation of species genetic diversity during evolution [38–40]. To sustain early embryo development and progress through stages of high TE expression, which normally induce strong stress and DNA damage responses, early-stage embryos undergo profound epigenetic remodeling. This includes developmentally programmed splicing failure that specifically attenuated DNA damage responses and suppressed p53 pathway activation [41,42]. In line with this, sustained activation of p53- and p16-dependent checkpoints, as hallmarks of senescent cells, strongly impairs both iPSC reprogramming efficiency and access to 2C-like states [9,14,15,43].

A growing understanding of totipotency *in vivo* as well as 2CLCs, which represent a small fraction (<1%) among ES and iPS cells when cultured under naïve conditions *in vitro* allowed to propose several approaches to move closer in establishing stem cell lines with totipotent potential [44,45]. Recent analysis of 2C embryos identified splicing failure as one of the features of the totipotent state and, in turn, spliceosome inhibition using different approaches appeared to be sufficient to generate stem cell lines with 2C-like potential [42]. In addition, analysis of ES cells under naïve culture conditions *in vitro* revealed that several factors, such as a pioneer transcription factor, Dux, epigenetic factors Tet and Setdb1, or a chemical cocktail to produce expanded pluripotent conditions (reviewed in [45]), all capable of regulating entry into a 2C-like state. Similar to 2CLC, the totipotent-like cells obtained in p16^High^ senescence-free conditions [9] express high levels of pluripotent genes, with a transcriptome and a landscape of enriched histone H3K4me3 and H3K27me3 markers at regulatory elements that more closely resemble ES cells than 2C embryos [46]. However, there were clear differences between totipotent-like cells obtained in p16^High^ senescence-free conditions [9] and 2CLCs, including the expression of Line1 retro-elements and Stella/Dppa3. Line-1 transcripts were found to be highly expressed and critical for regulation of global chromatin accessibility in the early mouse embryo [47]. In fact, the highest level of Line-1 transcripts was found in 2C embryos, with decreased levels by the 16C stage. Strikingly, this increase in Line-1 expression in early embryos does not induce any noticeable activation of DNA damage signaling, as assessed by γ-H2AX expression [47]. This could be partially explained by the suppression of the splicing machinery, as discussed above. In turn, the high Line-1 expression at early embryonic stages appeared to be essential for preimplantation development [47–49] and generation of somatic [50], and germline mosaicism [51] as a fundamental mechanism of genomic diversification. In a similar manner, Stella/Dppa3 is critical for early embryonic development, while its deletion results in significant attenuation of expression of MERVL in 2C embryos [52]. Thus, in addition to up-regulation of several 2C factors, including Dux and MERVL typical of 2CLCs, totipotent-like cells obtained in p16^High^ senescence-free conditions [9] express Line1 and Stella/Dppa3 to further reinforce totipotent state. Collectively, these observations suggest that totipotent-like cells generated in the absence of p16^High^ senescent cells share core transcriptional features with classical 2CLCs while exhibiting additional characteristics associated with enhanced developmental plasticity and early embryonic chromatin remodeling (Table 1).

**Table 1:** Comparison between classical 2C-like cells and totipotent-like cells generated in the absence of p16^High^ senescent cells

Feature	Classical 2CLCs	p16^High^-deficient totipotent-like cells
Dux expression	High	High
MERVL activation	High	High
Line-1 expression	Moderate/transient	High
Stella/Dppa3	Low/variable	High
SAM levels	Elevated	Elevated
Chromatin state	2C embryo-like	Intermediate ES/2C-like
Extraembryonic potential	Partial	Enhanced

The involvement of p16^High^ senescence in regulating totipotency appeared to be specific to the reprogramming context and does not mirror normal embryogenesis. In fact, p16^High^, in contrast with p21^High^ senescent cells, are absent during embryonic stages and appear only in adult animals [53,54]. Similarly, NNMT is expressed in the late stages of embryogenesis, while embryonic development is not impacted in NNMT knockout mice [11]. Furthermore, we found no evidence of the presence of p16^High^ cells in established cultures of iPS lines maintained under different conditions, including naïve conditions. The role of p16^High^ senescence appears to converge with the regulation of totipotency during early embryogenesis as a mechanism to control SAM levels, the universal donor of methyl groups. Earlier studies showed the highest level of SAM in 2C embryos *in vivo*, which is consistent with an enhanced demand for epigenetic regulation at this embryonic stage, including ZGA [55]. Furthermore, SAM levels seem to increase in the naïve 2CLC-containing state when compared with primed ES cells [56]. Our recent work further found that senescence- and SAM-dependent regulation of pluripotency only exists during iPS reprogramming, while extended culturing of established iPS cell lines or ES cells in the presence of senolytic drugs dasatinib and quercetin does not induce the appearance of totipotent properties [9].

Noticeable differences also emerge between *in vitro* and *in vivo* reprogramming. As discussed earlier, *in vitro*, p16^High^ cells arise prior to Nanog-positive iPSC, whereas *in vivo* reprogramming in young organisms proceeds without senescence induction, with p16^High^ cells appearing only during teratoma formation. As such, *in vivo* reprogrammed Nanog-positive iPSC should always be totipotent [57,58]. Indeed, capturing early *in vivo* OSKM-reprogrammed cells from blood and subsequently culturing them *in vitro* has been shown to produce iPSC with totipotent potential [57]. In contrast, when reprogrammed Nanog-positive cells start to produce teratomas *in vivo*, emerging p16^High^ senescent fibroblasts quickly derail the totipotent potential as the frequency of finding differentiated cells expressing extraembryonic markers Cdx2 and Elf5 is significantly lower or even absent from mice containing p16^High^ cells when compared with mice without such cells [9].

Beyond reprogramming, modulation of SAM availability emerges as a general regulator of cell fate plasticity. Experimental depletion of SAM or reduction of the SAM/SAH ratio markedly attenuates lineage transitions, exemplified by impaired BMP4-driven activation of trophoblast markers during blastoid differentiation [9]. These findings suggest that elevated activity of certain methyltransferases, such as NNMT, and reduced SAM availability, whether driven by senescence-dependent or independent mechanisms, can broadly restrict plasticity across developmental and regenerative contexts. Collectively, these data extend the p16^High^ senescence framework by positioning NNMT-SAM metabolism as a metabolic epigenetic axis that links senescence, reprogramming efficiency, and access to totipotent-like states. Targeting this axis may therefore represent a powerful strategy not only to enhance iPSC reprogramming and developmental potential, but also to restore plasticity and function in aged tissues and organisms [9,59].

## Partial reprogramming, epigenetic rejuvenation, and p16^High^ state

The concept of partial or transient reprogramming emerged from early observations that several hallmarks of cellular aging can be reversed without impacting somatic cell identity. Short-term expression *in vivo* in old animals, usually for 1–3 days, of the Yamanaka factors, most commonly OSK or OSKM, restores youthful epigenetic features in aged cells, including DNA methylation patterns, heterochromatin organization (such as H3K9me3 distribution), mitochondrial function, and global transcriptomic profiles [60,61]. These findings demonstrate that aspects of cellular aging are epigenetically reversible without complete dedifferentiation into pluripotent states.

*In vivo* studies provide compelling support for the rejuvenating potential of transient reprogramming. Cyclic OSKM expression in progeroid and naturally aged mice improves tissue regeneration, reduces inflammatory gene expression, and extends lifespan when tightly controlled to avoid excessive dedifferentiation [62–68]. These interventions restore aspects of youthful epigenetic organization and improve physiological parameters across analyzed tissues. Importantly, transient short-term reprogramming does not increase tumor incidence, suggesting that rejuvenation can be separated from oncogenic transformation within a defined therapeutic window [64–66,69–73]. Nevertheless, it should be kept in mind that the increased mutation burden commonly observed with aging could significantly accelerate eventual tumor formation [37,74]. Therefore, identifying *in vivo* cell types whose targeting would minimize such risks is of paramount importance in the field of partial reprogramming.

With respect to senescent cells, an important question is whether such cells, and particularly those expressing high levels of p16, remain amenable to epigenetic rejuvenation and to what extent they influence the responsiveness of neighboring cells to partial reprogramming signals *in vivo* in aged organisms. Our recent work indeed showed that p16^High^ cells strongly block the effects of partial reprogramming on maintaining blood vascularization and reducing fibrosis in old mice. In turn, continuous removal of p16^High^ cells significantly improved the impact of partial reprogramming on these key rejuvenation-associated histological features in the liver. This raises an important consideration regarding the therapeutic potential of targeting p16^High^ senescent cells in aged organisms as a strategy for rejuvenation [9].

Accumulating evidence suggests that partial reprogramming is most effective in cells that have not yet entered a deeply entrenched senescent state characterized by high expression of p16. In such cells, age-associated epigenetic alterations remain sufficiently plastic to permit reversal. This may also be the case in fibroblasts obtained from older individuals, where a fraction of cells does not express p16 at high levels and is therefore amenable to reprogramming [75]. In contrast, cells that have transitioned into a stable p16^High^ senescent state frequently exhibit persistent DNA damage signaling, large-scale chromatin reorganization, and stable transcriptional rewiring that collectively impose substantial barriers to complete epigenetic resetting. These observations suggest the potential existence of a functional epigenetic threshold beyond which rejuvenation becomes progressively more difficult and potentially irreversible [9].

Recent work has further refined this concept by demonstrating that rejuvenation can be targeted specifically to p16^High^ cell populations. In a recent study, targeted expression under the control of a fragment of the Cdkn2a promoter induced partial reprogramming in p16-positive cells in progeroid and naturally aged mice [62,76]. Remarkably, this strategy reduced inflammatory gene expression, improved wound healing, shifted hematopoietic stem cell composition toward a more youthful profile, and extended lifespan without increasing tumor formation.

These results may appear counterintuitive given the limited capacity to reprogram p16^High^ fibroblasts, as discussed above, and may have several potential explanations. First, they may suggest that some *in vivo* senescent cell types expressing high levels of p16 indeed retain a degree of epigenetic plasticity when reprogramming signals are precisely targeted and temporally controlled. Another explanation could stem from the fact that p16 is expressed in multiple immune cell types during aging, and the observed benefits of OSK-mediated partial reprogramming may occur in these cells. The latter possibility is particularly relevant, since p16^High^ immune cells do not necessarily enter a state of full senescence and may therefore remain amenable to the effects of partial reprogramming [11]. This raises the possibility that transient reprogramming interventions may preferentially rejuvenate immune-associated inflammatory programs without requiring full dedifferentiation. Such an approach could restore tissue-supportive immune functions while minimizing the oncogenic risk associated with extensive reprogramming of highly proliferative somatic cells. Because immune cells also orchestrate tissue inflammation, repair, and regeneration, selective rejuvenation of defined immune populations may exert systemic effects extending beyond the targeted cells themselves, potentially improving tissue repair, inflammatory balance, and organismal resilience during aging. Identifying such senescent and senescent-like cell types that are most responsive to partial reprogramming could therefore raise the possibility that their direct targeting may yield noticeable rejuvenation benefits in aged organisms.

Taken together, current evidence supports a model in which partial reprogramming operates within a narrow therapeutic window that enables rejuvenation while preserving cellular identity. The p16^High^ senescent state has traditionally been viewed as a critical boundary limiting this process, as cells that have not yet reached this state generally remain more responsive to rejuvenation signals. In contrast, deeply senescent cells exhibit extensive chromatin remodeling and stable transcriptional changes that can constrain epigenetic resetting. However, recent studies indicate that this barrier may not be absolute. Targeted expression of reprogramming factors in some senescent cells, as well as in cells undergoing DNA damage, has demonstrated that these cells can retain a degree of epigenetic plasticity when reprogramming cues are delivered in a controlled, cell-type-specific manner [77]. These findings in turn suggest that selective rejuvenation of p16^High^ cells may represent a feasible strategy to restore tissue function in aged organisms. Understanding how senescence-associated chromatin landscapes modulate responsiveness to reprogramming will therefore be critical for developing effective regenerative interventions based on partial reprogramming.

Senolytic therapies, which selectively eliminate senescent cells, have emerged as promising interventions for improving certain age-related conditions [78]. In the context of cellular reprogramming, senolytic interventions may enhance cellular plasticity by removing p16^High^ senescent cells that impose epigenetic, and metabolic barriers to rejuvenation and acquisition of youthful cellular states [9,79]. However, senolytic approaches also present important conceptual and therapeutic challenges. Several senolytic compounds impair early phases of reprogramming independently of senescent cell clearance, likely because these agents target stress-adaptive survival pathways that are transiently required during the acquisition of plasticity [34–36]. Moreover, accumulating evidence indicates that senescent populations are highly heterogeneous and can exert context-dependent effects on tissue regeneration, wound healing, and cellular plasticity, suggesting that future interventions may require selective targeting of specific senescence-associated states rather than broad elimination strategies [11,80]. Thus, combining transient partial reprogramming with precisely timed and mechanistically tailored senolytic interventions may represent a promising therapeutic strategy to maximize rejuvenation while minimizing oncogenic transformation, tissue dysfunction, and loss of cellular identity.

## Conclusions

p16^High^ senescence emerges as a regulator of cellular plasticity, acting as an active, epigenetically and metabolically enforced barrier to reprogramming, rejuvenation, and totipotency. Partial reprogramming exploits a window of epigenetic malleability that exists prior to entry into this deeply entrenched state, while senescence removal or metabolic rewiring can further expand accessible cell fate states.

Integration of senescence biology, partial reprogramming, tissue regeneration, and cellular plasticity research supports a unifying model in which rejuvenation is achieved not by erasing cell identity wholesale, but by selectively dismantling senescence-associated transcriptional, epigenetic, and metabolic constraints. Future therapeutic strategies will likely combine targeted partial reprogramming with senescence-modulating and metabolic interventions to maximize regenerative benefit while minimizing oncogenic risk.

## Open Access

Open access for this article was enabled by the participation of INSERM in an all-inclusive Read & Publish agreement with Portland Press and the Biochemical Society.

## Abbreviations

2CLC: 2C-like cell
iPSCs: inducing pluripotent stem cells
OSKM: Oct4, Sox2, Klf4, and Myc
SASP: senescence-associated secretory phenotype
TEs: transposable elements
ZGA: zygotic genome activation
